# Abstracts and Selections

**Published:** 1909-04

**Authors:** 


### Abstracts and Selections.

### Lengthening Human Life In This Country One-Third.

AN EPITOME OF PROFESSOR IRVING FISHER'S RFPORT AS A MEMBER OF THE NATIONAL CONSERVATION COMMISSION.

In President Roosevelt's message to Congress, one of the most striking features was the recommendation that we should conserve not only our material, but also our vital resources. As he said, we must remove the reproach that this nation takes more care for the lives of its hogs and cattle than for the lives of human beings.

What might be accomplished by a determined effort to conserve human life, has been estimated for the Conservation Commission by one of its members, Professor Irving Fisher, of Yale University. By the aid of eighteen medical experts, he has calculated that the prevention, even in a moderate degree, of preventable diseases, would lengthen human life in this country fully one-third and possibly much more.

It used to be supposed that the average length of human life was fixed by an iron law, and that, although the individual might occasionally elude the grim reaper for a time, the race as a whole must needs conform to this inexorable law of mortality. Such an idea originated from the fact that the early mortality tables for different countries showed a remarkable resemblance. Professor Fisher points out, however, that this resemblance between the mortality rates of different countries was due to the similarity among them of the conditions to which human life was exposed. More recent statistics show that the mortality rate varies greatly at different times and places. Statistics recently calculated for India show the startling fact that the average duration of life there is less than twenty-five years, as compared with fifty-two years for Sweden, or more than double. Besides this, it appears that life is rapidly lengthening in those countries where sanitary science and preventive medicine are being put in practice, while in India human life is not lengthening at all. It remains stationary, because so little progress has been made in the application of preventive medicine. In Europe, statistics show that the average length of human life has doubled in three and a half centuries (from 20 to 40 years) and that it is now increasing faster than ever. During the seventeenth and eighteenth centuries, life increased at the rate of about four years for each century. During the first half of the nineteenth century, it increased at the rate of about nine years per century. During the latter half of the nineteenth century the rate of increase was seventeen years per century and in Prussia, where medical and sanitary science has reached its highest development, it has increased at the rate of twenty-seven years per century. In this country, so far as can be estimated from the meager statistics (which are mostly from the State of Massachusetts), life is lengthening at about the rate of fourteen years per century, or half the Prussian rate.

But Professor Fisher, who has made these calculations, points out that we do not have to wait one hundred years to enjoy this increase. *Such a new lease of life, if we will, we may have for ourselves and our children.* All that is needed is to apply the known rules of prevention which medical experts have developed. More than three-fourths of the deaths from tuberculosis, which alone number 150,000 per year, are unnecessary. More than four-fifths of the deaths and illness from typhoid fever could be avoided. In Lawrence, Mass., after a pure water supply was installed, the deaths from typhoid shrank by eighty per cent.

Recent investigations by Professor Sedgwick and others show that for every death from typhoid prevented by sanitary measures, two or three deaths from other diseases are prevented at the same time. Professor Fisher calculates that pure water, pure milk, and pure air, if used throughout the nation to the extent that they are used in certain individual places would alone lengthen life by an average of eight years. He also estimates that there is a great amount of needless illness. There are at all times about three million people in the United States seriously ill, of whom about 500,000 are consumptives. Most of this illness is preventable. None of these calculations take account of discoveries in medicine or sanitary science calculated to lengthen human life, such as those which Professor Metchnikoff is endeavoring to perfect. Professor Fisher's figures are based merely on knowledge already gained and tested.

If we attempt to put in dollars and cents the gain which would come from the increase of human life in this country from the forty-five years, which is its present average, to the sixty years which statistics show could easily be attained, the results are startling. Reckoning the value of each life saved at only $1,700 and the value of the earnings lost by illness at only $700 per year, during the working period of life, it is found that over one and one-half billions of dollars is the money value of the lengthening and strengthening of life and efficiency which could be secured.

In order to obtain this result, co-operation is needed between individuals, cities, the States, and the national government. The health service of the nation would have to be improved and much more liberally supported. First of all, the health agencies of the government would need to be concentrated into one department, and this department must be so organized as to carry on a continuous campaign of education to teach people how to prevent disease and death. The Department of the Interior already contains a nucleus for such concentration in the Bureau of Education and in its control of certain government hospitals. The most important agency, however, is the Public Health and Marine Hospital Service, now in the Department of the Treasury, with which it is entirely incongruous, while the administration of the Pure Food and Drugs Act is now in the hands of the Department of Agriculture. That such health agencies should be concentrated is the recommendation of President Roosevelt, and this is known to accord with the wishes of President-elect Taft, and was also introduced by special planks into the platforms of both political parties. Such a movement has for a long time been advocated by the American Health League and the Committee of One Hundred on National Health, and for a still longer time by the American Medical Association.—*American Health.*

---

THE DIFFERENTIATION between a specific and tuberculous ulcer of the fauces is sometimes very difficult. As a rule the specific ulcer is shallow, grayish, with a regular margin, not very tender and does not cause dysphagia; on the other hand, a tuberculous ulcer is deeper, more sloughy, irregular in outline, has an outer inflammatory zone, is exquisitively tender and causes great pain on swallowing; laryngeal examination may reveal a tuberculous condition of the cords.—*American Journal of Surgery.*

---

### Alcoholic Heredity.

The following quotation is taken from a paper by Dr. Cohan, of Anna, Illinois, which appeared in the *Journal of the American Medical Association:*

"It has been disputed whether alcoholism is the effect or the cause of many neuropathic and psychopathic conditions. Both sides of the question can be answered in the affirmative. In many cases alcoholism is one of the most evident of psychopathic traits. We should look on the drunkard, not always as a creature who indulges himself in a vice, but as one chained to a habit through the shortcomings of his ancestors. For the real cause of much of the drunkenness in our land lies even deeper than the existence of the liquor traffic.

"Alcoholism is undoubtedly productive of numerous forms of degeneracy, and investigation of the history of those patients received by insane hospitals, as taken from the most authentic records of Europe and this country, show that one-third to one-fourth of the total number have ancestors who were affected by alcoholism. Practically the percentage holds good in the history of asylums for the feeble-minded and epileptics. Although to the casual observer the habitual drunkard, between periods of intoxication, may appear approximately normal, in time more or less degenerative changes take place in his mental and moral character, betraying themselves in the failing sense of all ethical impulses, and causing him gradually to descend the moral scale, even, perhaps, to depravity and crime; and eventually his wakened mental functions may render him an easy victim of hopeless lunacy.

"Much said regarding alcoholism applies also to the different drug habits.

"Degenerative defects of all kinds, usually coupled with ignorance, and often productive of insanity and kindred diseases, are found in great numbers among prostitutes, and are likewise of frequent occurrence among all criminal classes and their descendants. We must conceive that the habitual criminal, like the habitual drunkard, is often, unhappily, an expression of defective mentality, a victim of transmitted tendencies whose rightful place can not with certainty be pronounced to be the penitentiary."—*Journal Inebriety.*

---

### The Passing of Another Delusion.

BY DAVID PAULSON, M. D.

A little more than a generation ago almost everybody labored under the delusion that in order to be strong one must drink liquor. The average mortal saw to it that his cellar was well stocked with stimulating drinks before the winter set in. That delusion has died hard, but it is dying, just the same.

Another delusion that we have harbored almost as persistently is the deeply rooted notion that in order to be strong we must eat a large amount of flesh food. But light is breaking in upon the minds of the brightest lights in the medical profession and of the most sensible men and women, that overloading the system with proteid food has been the cause of more Bright's disease, rheumatism, nervous prostration, and mental breakdown than was generally supposed. We quote the following editorial from the *Ladies' Home Journal:*

"The fact that we are eating less meat, as a nation, is a tribute to our common sense and a good thing for our health. We have lived all too long under this meat delusion in America. It is not so long ago that the steak or chop at breakfast was a usual sight; now it is a rarity at well-considered tables. Men's luncheons are getting simpler, and the three-times-a-day meat idea is rapidly changing for the meat-once-a-day rule.

"A curious fact is that men are clinging with a greater tenacity than are women to the idea that meat more than once a day is necessary for strength. Years ago nursing mothers exploded the idea for themselves; many a woman found that to eat meat three times a day did the child at her breast more harm than good; it made the child restless and failed to give lasting nourishment. And this latter point, so incontrovertibly proved by women, is what men cannot get through their heads. The wise little Japanese found out this truth centuries ago, and his endurance is marvelous. Some day the American man will find it out, and when he eats less meat he will be the better for it."

That our people have begun to discover that there is better food for health and strength and mental and moral clearness than feeding on animal flesh is shown from a recent report of the United States Department of Agriculture, which states that meat eating is slowly decreasing in the United States, although compared with other countries in 1904 there was eaten in this country 185 pounds of flesh food for every man, woman and child in it, while in Great Britain there was used only 121; in France, 79; in Belgium, 70; in Denmark, 76; in Sweden, 62; in Italy, 46.—From *Good Health.*

---

THE Philadelphia County Medical Society has presented a bill to the Legislature which makes a provision forbidding all physicians from practice who are addicted to the use of liquor and drugs. Where this fact can be proved the license is to be revoked. Should a cure be effected, the physician may be reinstated; but for the second offense the license is to be revoked permanently.

---

### Dr. Lydston Gains Allies in Fight on Dr. Simmons.

Aims New Blow at Editor and Wins 196 out of 200 Present at North Shore Meeting.

Dr. G. Frank Lydston, who has been publicly criticising Dr. G. H. Simmons, Secretary of the American Medical Association, and editor of its official publication, grilled him in merciless manner last night before the North Shore branch of the organization at the Bismarck garden. Then he asked all those present who believed in his course to rise, and all but four of the 200 doctors accepted the invitation.

Dr. Simmons was not present. It was reported he had been called out of the city.

Prof. C. S. N. Halberg, of the Chicago Pharmacy School held that although Dr. Simmons might have been unethical years ago he should be given credit for what he had accomplished for the Association.

ACCUSES, THEN RETRACTS.

"When Dr. Hamilton died nine years ago the trustees had difficulty in getting a proper editor for the *Journal*," he said, "and they had to go to Lincoln, Neb., and get Dr. Simmons."

"Some of the prejudice against him might be due to jealousy. I remember how certain gentlemen had their lightning rods up expecting to be struck——"

"Do you mean to insinuate that I was an applicant for the job of editor of the *Journal*?" broke in Dr. Lydston, hotly.

"Yes, my recollection is that you had your lightning rod up," replied the speaker.

"Well, I insist on you retracting that statement, for it is unequivocally and maliciously false," shot back Dr. Lydston. "You can not get away with that statement before this audience."

The speaker hesitated and looked confused and then said:

"I guess I am mistaken about that."

Dr. Lydston also took a couple of sharp jabs at Dr. S. C. Stanton, associate editor of the *Journal*, who was expected to make a sharp reply. Instead, however, Dr. Stanton confined himself strictly to the division of fees phase of the case.

ATTACKS DR. SIMMONS' RECORD.

Dr. Lydston told how his scheme to have his ideas printed in the *Journal* was frustrated and said the publication of two pamphlets was the only way open to show the doctors generally the history of Dr. Simmons' private hospital at Lincoln, Neb., twenty years ago.

"I'm the goat, but I've got a few butts left," he said, "and your committee on 'whitewash or persecution' can do as it sees fit. Dr. Simmons was head of one of the biggest fakes that ever has existed. Just see these old-time advertisements of his. Here is one reading, 'A limited number of lady patients can be accommodated at my home.'

"If any one present thinks Dr. Simmons is the kind of a man you want in your association, please arise," requested Dr. Lydston. The audience remained seated, and then Dr. Lydston said:

"Now, then, all who approve the course I took in advertising Dr. Simmons' record please arise," and all but four stood.

Loud applause greeted this show of strength of the warring factions.—*Chicago Tribune*, April 8, 1909.

"Our national health is physically our greatest asset. To prevent any possible deterioration of the American stock should be a national ambition. We can not too strongtly insist on the necessity of proper ideals for the family, for simple living, and for those habits and tastes which produce vigor and make men capable of strenuous service to their country. The preservation of national vigor should be a matter of patriotism. Federal activity in these matters has already developed greatly, until it now includes quarantine, meat inspection, pure food administration and Federal investigation of the conditions of child labor. It is my own hope that these important activities may be still further developed."—*Theodore Roosevelt.*

